# Dr. J. B. S. Jackson on the Comparative Frequency of Tuberculous Disease

**Published:** 1842-07-23

**Authors:** 


Dr. J. B. S. Jackson, who is well known to the profession as an ardent cultivator of pathological anatomy, has given us the results of his observations as to the "Comparative Frequency of Tuberculous Disease."

***Comparative Frequency of Tuberculous Disease.*** By J. B. S. Jackson, M. D.—The following is an analysis of dissections made during the last ten years in this city or the immediate vicinity, and tending to confirm the general statement, that in the temperate latitudes about one-sixth, or more, of our race die from some form of tuberculous disease.

The whole number of autopsies I find to be 604.

Of these there were excluded 94 cases of patients dying from disease foreign to the lungs, and in which these organs were not at all, or not sufficiently, examined; also four cases in which there was a question between pneumonia and tuberculous disease.

Of phthisis there were 93 cases. Amongst these were included some cases of general tuberculous disease in children, and in which some of the other organs may have been as much, or more affected than the lungs. One of these last was a child that died of tuberculous disease of the brain, with considerable disease of the bronchial glands, and only a trace in the lungs.

Acute meningitis in 16 cases, tuberculous disease being found in all of them, either in the lungs, the bronchial glands, or in both. The disease was undoubtedly tubercular, though in the first four cases reported, granulations are not mentioned as having been found in the membranes of the brain, not being acquainted, at that time, with the true nature of the disease.

A case of tubercular peritonitis, also, may be mentioned, in which, though the bronchial glands were considerably diseased, a single granulation only was found in the lungs.

In the above 604 cases, then, death is supposed to have been caused by tuberculous disease in 110, or in about one in $5\frac{1}{2}$.

In the remaining 396 cases, death was caused by some other than tubercular disease; but in all, the lungs were examined, and in a large proportion it is expressly stated whether there was, or was not found any such disease. Without doubt, the existence of tuberculous disease was occasionally overlooked, but, on the contrary, it was often noted when found in a very small or even minute quantity.

In the above 396 cases, there was no tuberculous disease nor any remains of any found in 306; the wilted appearance, so often met with at the apex of the lungs, not being considered as satisfactory evidence of the disease having formerly existed. The existence of cretaceous matter, however, was so considered, and cases where this was found were noted as tuberculous, without any discrimination. In 46 cases, tubercles were found it the lungs alone, and in 21 in the lungs and bronchial glands. In 20 they existed only in the bronchial glands, and were in the cretaceous form in all except two. In two cases there were cretaceous masses in the upper part of the thorax, supposed to be the result of old tuberculous disease, though there was no appearance of this last in the lungs, nor in the bronchial glands; neither was there in another case in which an extensive cretaceous deposit was found in the renal capsules, and which was similarly explained.

Several years since my attention was directed to the subject of the infrequency of the occurrence of the tubercular deposit in patients dying from malignant disease, and I was not aware, for some time, that the same remark had been made by others. Of 33 cases of malignant disease, in nearly all of which a careful examination was made for tubercles, it is expressly stated that in 24 none were found; six times they were found in the lungs alone, and in the bronchial glands alone three times.

Of 35 patients dying of various diseases, all of whom were decidedly intemperate, and most of them grossly, in 26 no tubercles were found; in five there were tubercles in the lungs; in one in the bronchial glands; in one in the lungs and bronchial glands; and only two died of phthisis. In several of the most striking, the organs were as free from tuberculous disease as those of a new-born infant. All of these 35 are stated, in the recorded history of the case, to have been intemperate; probably others are so recorded, and the list might have been considerably increased; but no case has been referred to under this head, except where I happened to remember that the patient had been intemperate.

Intemperance certainly does not seem to develope tubercles, even if it has not some effect as a prophylactic; the remedy, to be sure, would be worse than the disease; but has it any such effect?—*New England Quarterly Journal of Med. and Surg. July*, 1842.

The last remark, as to the comparative rarity of tuberculous disease in confirmed drunkards, has often occurred to us while in attendance upon a public institution in which a large number of very intemperate people are admitted. In conversation with the resident physicians at the visit, this fact was often alluded to, and the correctness of it was generally admitted. Long continued drunkenness produces a different class of diseases from phthisis; that is, of the heart, liver, kidneys, and of the brain. Individuals who are predisposed to phthisis are, however, often victims to intemperance, which proves fatal to many tuberculous subjects; but if they resist the first injurious results of their habits, they fall into a different condition from that of tuberculous subjects, become bloated rather than emaciated, and suffer from another set of symptoms.

